# Pinostilbene Hydrate Inhibits the Migration and Invasion of Human Nasopharyngeal Carcinoma Cells by Downregulating MMP-2 Expression and Suppressing Epithelial–Mesenchymal Transition Through the Mitogen-Activated Protein Kinase Signaling Pathways

**DOI:** 10.3389/fonc.2019.01364

**Published:** 2019-12-03

**Authors:** Pao-Yu Tseng, Yen-Tze Liu, Chia-Chieh Lin, Yi-Ching Chuang, Yu-Sheng Lo, Yi-Ting Hsi, Ming-Ju Hsieh, Mu-Kuan Chen

**Affiliations:** ^1^Department of Otorhinolaryngology, Head and Neck Surgery, Changhua Christian Hospital, Changhua, Taiwan; ^2^Department of Family Medicine, Changhua Christian Hospital, Changhua, Taiwan; ^3^Institute of Medicine, Chung Shan Medical University, Taichung, Taiwan; ^4^Department of Holistic Wellness, Mingdao University, Changhua, Taiwan; ^5^Oral Cancer Research Center, Changhua Christian Hospital, Changhua, Taiwan; ^6^Graduate Institute of Biomedical Sciences, China Medical University, Taichung, Taiwan

**Keywords:** pinostilbene hydrate, MMP-2, epithelial–mesenchymal transition, MAPK, nasopharyngeal carcinoma

## Abstract

**Background:** Nasopharyngeal carcinoma (NPC) is one of the most common head and neck cancers in East and Southeast Asia. During the past decades, advances in radiotherapy and chemotherapy had shown the improvement in tumor control with fewer side effects. Nevertheless, metastasis of NPC causes treatment failure and is often associated with poor clinical outcome and cancer mortality.

**Hypothesis/Purpose:** Pinostilbene hydrate (PSH) was recently demonstrated to have anti-metastatic properties on human oral cancers. However, the effects of PSH on NPC cells remain unknown.

**Methods and Results:** This study aims to investigate the anti-cancer ability of PSH on human NPC by wound healing, transwell assays, zymography assay, and Western blot assay to explore the possible underlying mechanisms. PSH significantly reduced the migrated distance of NPC cells in a dose-dependent manner and the abilities of cancer cell migration and invasion were markedly inhibited. The activity and the expression of MMP-2 were also significantly decreased after treatment with PSH. Furthermore, combined treatment of PSH with ERK1/2 inhibitor (U0126) caused significant elevation of the activity and the expression of MMP-2. Additionally, PSH upregulated the expression levels of E-cadherin and Claudin-1 while downregulating that of N-cadherin and vimentin on both NPC cell lines.

**Conclusion:** Our research illustrates that PSH inhibits the migration and invasion of human NPC cells. After exposure to PSH on NPC, the expression of MMP-2 is downregulated and EMT is suppressed through MAPK signaling pathways. These observations suggest that PSH could be a potential anti-metastatic agent for patients with NPC.

## Introduction

Nasopharyngeal carcinoma (NPC), a malignant epithelial tumor arising from the lining of the nasopharynx, is commonly found at the fossa of Rosenmüller. It has a distinct geographical and ethnic distribution. Many of new cases were reported in east and southeast parts of Asia (1). Worldwide, an estimated 87,000 incident cases and 51,000 deaths occur annually, accounting for about 0.6% of the global cancer burden (1). In Taiwan, the age-adjusted incidence rate of NPC per 100,000 person-years is 8.18 for men and 2.35 for women (2), in contrast to <1 in North America and Europe for both sexes (1). Many risk factors are associated with NPC, including Epstein-Barr virus (EBV) infection (3), host genetic susceptibility in endemic regions (4–6), dietary exposure to salted fish and environment factors (7, 8). Depending on the histological appearance, the World Health Organization (WHO) subtypes NPC as keratinizing squamous cell carcinoma (SCC), non-keratinizing carcinoma and basaloid SCC. Non-keratinizing carcinoma is further sub-categorized into differentiated or undifferentiated tumors (9). In regions with endemic NPC, undifferentiated carcinoma comprises over 95% of cases and the vast majority are associated with EBV infection (10, 11).

During the past decades, advances in population screening, diagnostic imaging, intensity-modulated radiotherapy (IMRT) and systemic agents have driven the improvement in disease control and survival (12, 13). The primary treatment for early stage patients is definitive radiotherapy and the addition of chemotherapy for locally advanced cases has shown a benefit in overall survival (12, 14). However, the metastatic potentiality of NPC remains the major reason of treatment failure for patients; the 5- and 8-years distant metastatic rates were reported as 14.5 and 16.4% in the era of IMRT (15). Furthermore, around 15% of cases were found to have distant metastases at initial staging in endemic NPC (16). Therefore, an effort must be made to optimize the management of tumor invasion and migration in order to achieve a better clinical outcome.

Cancer metastasis is a complex process; it involves the translocation of a cancer cell to a distant organ and the colonization of tumor cells at distant site (17). The metastatic dissemination consists of a series of sequential, interrelated steps: cancer cells invade the host stroma and surrounding tissue, enter the lymphatic channels and blood systems (intravasation), survive in the circulation and translocate to distant organs through the bloodstream, exit from the bloodstream (extravasation), survive the foreign microenvironment of distant tissues, and finally proliferate within the organ parenchyma into a macroscopic metastatic tumor (colonization) (17, 18).

Invasion of ECM is another critical process in cancer metastasis; both chemokine signaling and interactions between cancer cells and physical components of the metastatic niche are involved (17, 19). The matrix metalloproteinases (MMPs), a family of zinc-dependent endopeptidases that consist of more than 20 human MMPs, are the most prominent proteinases associated with cancer progression. Numerous studies had found the upregulated activity of MMPs in head and neck SCC and a negative association between the MMP level and the prognosis (20–24). Therefore, to understand how MMPs work in metastasis of NPC may help us to find more ways of tumor control.

People are increasing their interests in studying naturally extracted compounds or their analogs as one of anti-cancer agents. Resveratrol (*trans*-3,5,4′-trihydroxystilbene), existing in grapes, berries and red wine, is one of the well-studied agents with potential roles in both cancer prevention and treatment (25, 26). Resveratrol also showed synergistic effects with 5-fluoruracyl and cisplatin in the adjuvant therapy which increased the chemosensitization of cancers (27, 28). While resveratrol is unstable in the environment, it is very sensitive to air and light. For years, many derivatives of resveratrol were investigated and studies demonstrated that methylated resveratrol derivatives were more effective in the treatment of cancer with better bioavailability and bioactivity (29–31). Pinostilbene (3,4'-dihydroxy-5-methoxystilbene) is one of the naturally occurring methylated derivatives of resveratrol; it was reported to exert a potent neuroprotective effect against parkinsonism mimetic 6-hydroxydopamine-induced neurotoxicity in SH-SY5Y cells (31) and inhibitory effects on human colon cancer cells (32). More recently, study also found the antimetastatic effects of pinostilbene hydrate (PSH) on human oral SCC by downregulation of MMP-2 through mitogen-activated protein kinase (MAPK) signaling pathway (33). However, anti-cancer ability of PSH on NPC cells remains unknown. Here we try to evaluate the effects of PSH on NPC-039 and NPC-BM cells and discuss the possible involved mechanisms.

## Materials and Methods

### Reagents

Pinostilbene hydrate (3, 4′-Dihydroxy-5-methoxy-trans-stilbene hydrate, PSH) (≥95% purity) was purchased from Sigma-Aldrich (St. Louis, MO, USA) and dissolved in pure grade dimethyl sulfoxide (DMSO). Antibodies against MMP-2, protein kinase B (PKB, also known as AKT), phospho-AKT (p-AKT), p38, phospho-p38 (p-p38), extracellular signal-regulated kinase 1/2 (ERK 1/2), phospho-ERK 1/2 (p-ERK1/2), c-Jun N-terminal kinase 1/2 (JNK 1/2), phospho-JNK1/2 (p-JNK1/2), E-cadherin, Claudin-1, Vimentin, and N-cadherin were purchased from cell Signaling Technology (Danvers, MA, USA) and stored at −20°C. β-actin was bought from Novus Biologicals (Littleton, CO, USA). 3-(4, 5-dimethylthiazol-2-yl)-2, 5-diphenyltetrazolium bromide (MTT) were obtained from Sigma-Aldrich (St Louis, MO, USA) and stored at 4°C. Specific ERK1/2 inhibitor (U0126) and JNK1/2 inhibitor (SP600125) were purchased from Santa Cruz Biotechnology (Santa Cruz, CA, USA) and stored at −20°C.

### Cell Culture

The human NPC cell lines NPC-BM and NPC-039 were provided by doctor Jen-Tsun Lin, Hematology and Oncology, Changhua Christian Hospital. The human nasopharyngeal normal primary cell line was obtained from Celprogen (Torrance, CA). The cells were cultured in RPMI 1640 Medium (Gibco BRL, Grand Island, NY, USA) supplemented with 10% fetal bovine serum (FBS), 1% penicillin/streptomycin, 1.5 g/l sodium bicarbonate, 1 mM glutamine, and 1 mM sodium pyruvate (Sigma, St. Louis, MO) at 37°C in one atmosphere with 5% CO_2_.

### Cell Viability (MTT Assay)

The cell viability was determined by MTT assay. NPC-039 and NPC-BM (1 × 10^4^ cells/well) were seeded in 24-well plates and treated with various concentrations of PSH (0, 20, 40, and 80 μM) for 24 h. Then the medium was removed and the cells were incubated with MTT reagent 500 μl (0.5 mg/mL) at 37°C in 5% CO_2_ for 4 h. After removing the supernatant, DMSO was added to dissolve the formed formazan crystals. At last, the absorbance of the converted dye was measured using an enzyme-linked immunosorbent assay (ELISA) plate reader at a wavelength of 595 nm to determine the cell viability.

### Wound Healing Assay

NPC-039 and NPC-BM cells were seeded on 6-well plates at 5 × 10^5^ cells/well and incubated for 24 h. Then the cells were wounded by scratching with a 200-μl sterile pipette tip. After being washed twice, the cells were treated with PSH (0, 20, 40, and 80 μM) for 24 h. Then the cell migration was observed and photographed at the appropriate fields by using a phase-contrast microscope. The wound closure was monitored for 6 h. The cell-free area in the dish was measured by an inverted microscope (OlympusIX71, Tokyo, Japan) and the cell migrated distance was calculated by ImageJ software. Experiments were performed for at least three times.

### Cell Migration and Invasion Assays

Cell migration and invasion assays were performed as previously described (33, 34). NPC-039 and NPC-BM cells were incubated with different concentrations of PSH (0, 20, 40, and 80 μM) for 24 h. For migration assay, the lower chamber was filled with 600 μL of medium supplemented with 10% FBS in advance. Then the treated cells were seeded to the upper compartment of Transwell (Greiner Bio-One, North Carolina, USA) and incubated at 37°C for 16 h. For invasion assay, the treated cells were seeded in a matrigel (25 mg/50 mL, 60 μl; BD Biosciences, MA) coated upper compartment of chamber with growth medium placed in underneath chamber and incubated for 24 h. The non-migratory cells were removed after incubation for 24 h. Finally, the membrane was fixed with methanol and stained with 10% Giemsa for 2 h. The migratory cell number was quantified by randomly counting at more than three independent visual fields under the light microscope.

### Assessment of MMP-2 Activity

NPC-039 and NPC-BM cells were plated in 24-well plates and treated with different concentrations of PSH (0, 20, 40, and 80 μM) for 24 h. Then the cultured medium was collected, mixed with a loading buffer and subjected to 0.1% gelatin−8% sodium dodecyl sulfate-polyacrylamide gel electrophoresis (SDS-PAGE). After the proteins were electrophoretically separated, the gels were washed for 30 min twice in 2.5% Triton X-100 and incubated in reaction buffer (40 mM Tris-HCL, pH 8.0, 0.02% NaN_3_, 10 mM CaCl_2_) at 37°C for overnight. Subsequently, the gel was stained with Coomassie Brilliant Blue R-250 as described previously (33, 34) and the zones corresponding to proteolytic activity of MMP-2 were analyzed by ImageJ software.

### Western Blot Analysis

The treated cells were harvested and lysed in ice-cold RIPA buffer to extract all proteins. The protein concentrations were detected using the bicinchoninic acid (BCA) assay. Equal amounts of proteins were separated by 10% polyacrylamide gel and transferred onto a polyvinylidene fluoride (PVDF) membrane (EMD Millipore). After blocking in 5% non-fat milk with TBS-T buffer (20 mM Tris, 137 mM NaCl, pH 7.4) at room temperature for 1 h, membranes were incubated with diluted primary antibody (dilution of 1:1,000) and β-actin at 4°C with gentle shaking for overnight. Then washing with TBS-T for three times, membranes were incubated with the appropriate horseradish peroxidase-conjugated secondary antibody at room temperature for 1 h, rewashing with TBS-T for three times, the blots were visualized using an enhanced chemiluminescence (ECL) reagent (EMD Millipore) and autoradiography. β-actin was used as a loading control. The relative photographic density was quantified by ImageQuant LAS 4000 mini Biomolecular Imager (GE Healthcare Bio-Sciences AB, Björkgatan 30 751 84 Uppsala, Sweden). The relative density was quantitated with gel documentation and analysis (AlphaImager 2000; Alpha Innotech Corporation, San Leandro, CA, USA).

### Statistical Analysis

Statistical analysis was performed using SigmaStat 2.0 (Jandel Scientific, San Rafael, CA). Data were presented as the mean ± standard deviation (SD) of at least three independent experiments. Student's *t*-test was used for comparison among different groups. *P* < 0.05 was considered as statistically significant.

## Results

### Effects of PSH on the Viability of Human NPC Cells

**Figure 1:**
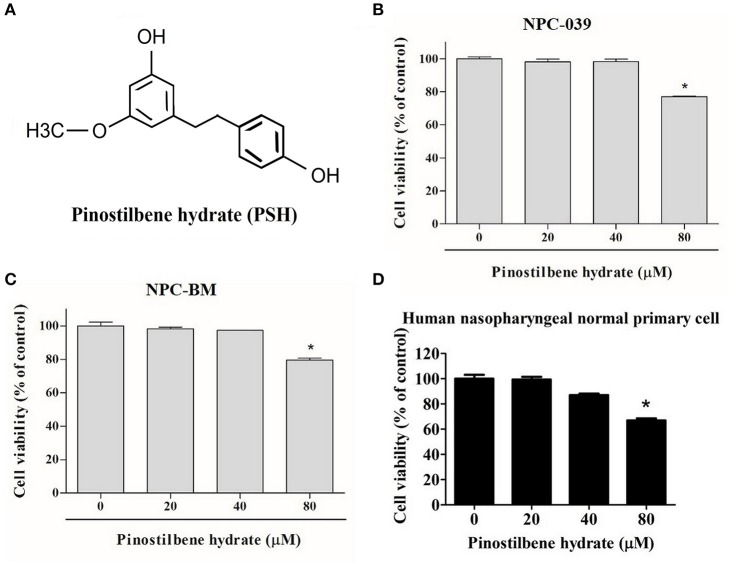
Effects of PSH on the viability of human NPC cells. NPC-039 and NPC-BM cells were treated with various concentrations of PSH (0, 20, 40, and 80 μM) for 24 h. The cell viability was determined by MTT assay. **(A)** Chemical structure of pinostilbene hydrate (PSH). The cytotoxicity was observed on **(B)** NPC-039, **(C)** NPC-BM, and **(D)** human nasopharyngeal normal primary cell lines. PSH significantly inhibited the cell viability of NPC-039 and NPC-BM cells after treatment with 80 μM of PSH. The values are the mean ± SD of at least three independent experiments. *P < 0.05.

To examine the cytotoxicity of PSH on NPC cell lines, the cells were evaluated by MTT assay. The chemical structure of PSH is shown in Figure 1A. NPC-039, NPC-BM, and human nasopharyngeal normal primary cell lines were treated with various concentrations of PSH (0, 20, 40, and 80 μM) for 24 h. The growth-inhibitory effects were observed (Figures 1B–D). Comparing with the control group, PSH significantly inhibited the cell viability when NPC-039 and NPC-BM were incubated with 80 μM of PSH. No significant cytotoxic effects were observed on both cell lines when treated with other concentrations of PSH. A concentration range of 0–80 μM of PSH was then chosen for subsequent experiments.

### Inhibitory Effects of PSH on Motility, Migration, and Invasion of NPC Cell Lines

**Figure 2:**
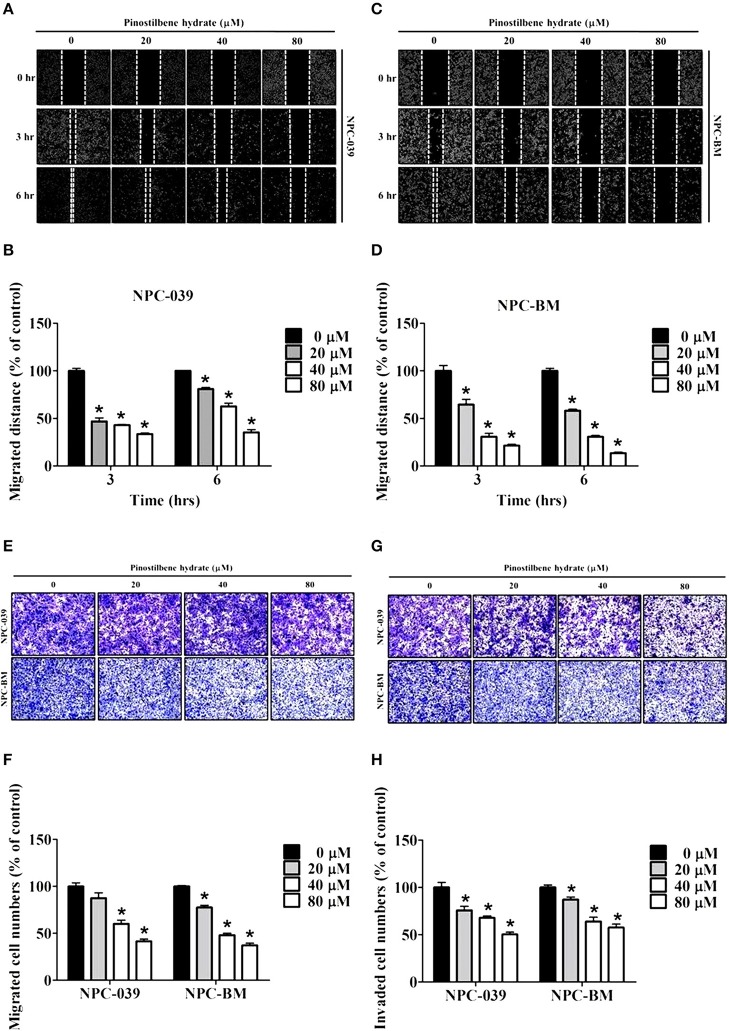
Effects of PSH on motility, migration, and invasion of NPC cell lines. NPC cells were treated with various concentrations of PSH (0, 20, 40, and 80 μM) for 24 h. The migrated distance was determined by the wound healing assay on NPC-039 **(A,B)** and NPC-BM **(C,D)** cell lines. PSH significantly reduced the migrated distance of NPC cells when pictured at 3 and 6 h in a dose-dependent manner. To evaluate the effects of PSH in migration **(E,F)** and invasion **(G,H)** transwell assays were used. The abilities of cell migration and invasion were markedly inhibited by PSH on both cell lines in a dose-dependent manner. The values are the mean ± SD of at least three independent experiments. *P < 0.05.

To determine the role of PSH on motility of NPC cells, the wound healing assay was performed. The results showed a significant reduction in migrated distance for both cell lines after exposure to PSH (Figures 2A–D). PSH markedly inhibited the wound gap closure in a dose-dependent manner. To evaluate the effects of PSH in migration and invasion of NPC-039 and NPC-BM cells, transwell assays were used. As shown in Figure 2, the abilities of cancer cell migration (Figures 2E,F) and invasion (Figures 2G,H) were significantly decreased by PSH on both cell lines in a dose-dependent manner. These results indicated that PSH played a negative role in migration and invasion of human NPC cells.

### PSH Downregulated MMP-2 Activity and Expression on NPC Cells

**Figure 3:**
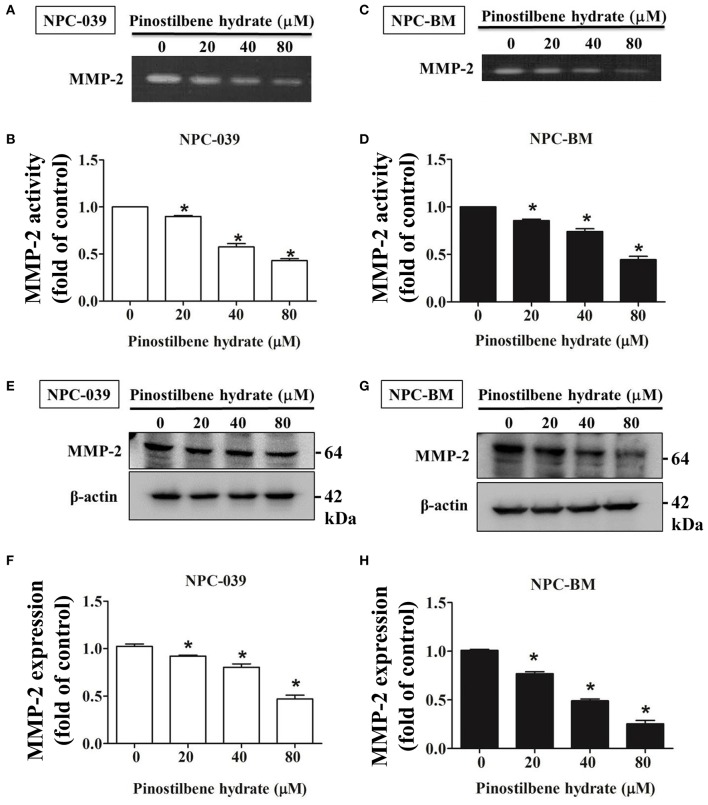
PSH downregulated the activity and expression of MMP-2 in NPC cells. The activity of MMP-2 in PSH treated human NPC cells was examined by the gelatin zymography assay. Significant decreased activity of MMP-2 in a dose-dependent manner was noticed on NPC-039 **(A,B)** and NPC-BM cell lines **(C,D)**. **(E–H)** The expression of MMP-2 level was detected by Western blot assay. PSH significantly reduced the expression of MMP-2 in a dose-dependent manner on both cell lines. β-actin was used as a loading control. The values are the mean ± SD of at least three independent experiments. *P < 0.05.

Degradation of ECM by extracellular proteinases is crucial to the invasion and migration of cancer cells (35, 36) and MMPs are the most famous proteinases associated with tumorigenesis. We investigated the activity of MMP-2 in PSH treated human NPC cells through the gelatin zymography assay. After exposure to PSH (0, 20, 40, and 80 μM) for 24 h, significantly reduced activity of MMP-2 in a dose-dependent manner was noticed on NPC-039 and NPC-BM cells (Figures 3A–D). Furthermore, the expression of MMP-2 was examined by Western blot assay. With higher dose of PSH administration on both cell lines, the expression level of MMP-2 became lower (Figures 3E–H). PSH significantly downregulated the activity and expression of MMP-2 in human NPC cells.

### PSH Reduced the Activity of MMP-2 via the MAPK Signaling Pathways on NPC-039 and NPC-BM Cell Lines

**Figure 4:**
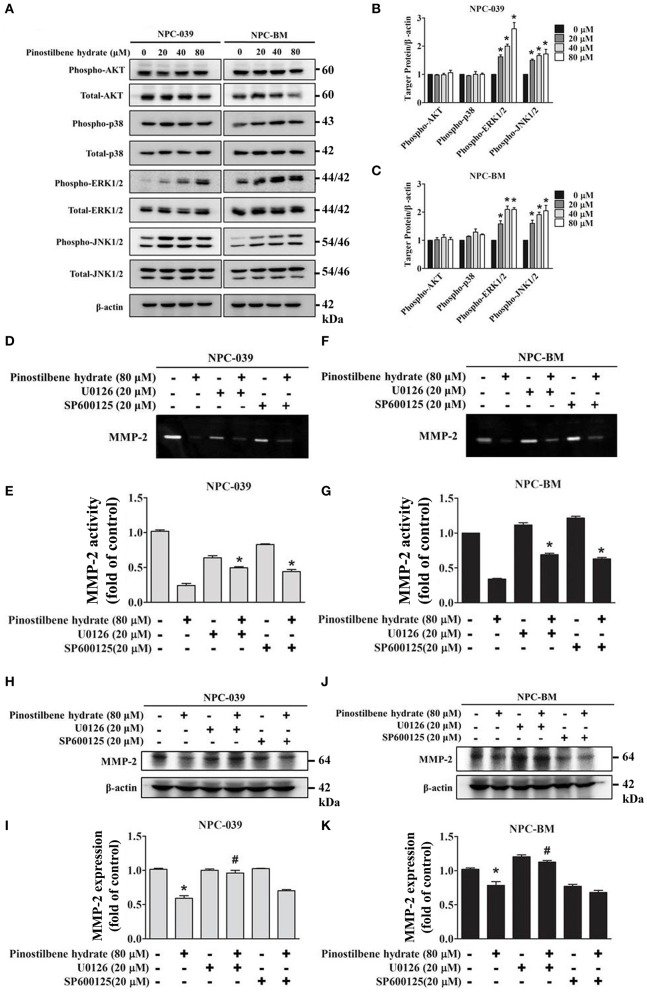
PSH regulated the activity of MMP-2 via the MAPK signaling pathways on NPC-039 and NPC-BM cell lines. **(A)** Cells were treated with different concentrations of PSH (0–80 μM) for 24 h. The p-AKT, p-ERK1/2, p-JNK1/2, and p-p38 were analyzed by Western blot with their respective antibodies. The total protein of AKT, ERK1/2, JNK1/2, and p38 is also shown. **(B,C)** PSH significantly promoted the expression of p-ERK1/2 and p-JNK1/2 on NPC-039 and NPC-BM cell lines in a dose-dependent manner. Values represent the mean ± SD of at least three independent experiments. ^*^P < 0.05, compared with the control (0 μM). **(D–G)** The cells were pretreated with or without ERK1/2 inhibitor (U0126) or JNK1/2 inhibitor (SP600125) followed by treatment with PSH (0 and 80 μM) for 24 h significantly upregulated activity of MMP-2 in combined treatment of PSH with U0126 or SP600125 was noted. ^*^P < 0.05, compared with 80 μM PSH treated only group. **(H–K)** A co-treatment of PSH with U0126, but not SP600125, significantly contributed to the elevated expression of MMP-2 level on both cell lines. ^*^P < 0.05, compared with the control. ^#^*P* < 0.05, compared with 80 μM PSH treated only group.

Previous studies indicated that the MAPK pathways were involved in the regulation of MMP-2 expression on oral cancer cells (33, 37). To evaluate the associated mechanisms in NPC, we directly measured the expression and the phosphorylation of AKT, ERK1/2, JNK1/2, p38 on NPC cells in response to PSH by Western blot analysis. The findings revealed that PSH promoted the expression of p-ERK1/2 and p-JNK1/2, but not p-AKT and p-p38, on NPC-039 and NPC-BM cell lines in a dose-dependent manner (Figures 4A–C). To further clarify whether PSH regulated the activity of MMP-2 through MAPK signaling pathways, cells were pretreated with or without specific ERK1/2 inhibitor (U0126) or JNK1/2 inhibitor (SP600125) for 1 h followed by treatment with PSH (0 and 80 μM) for 24 h. The activity of MMP-2 was then determined by gelatin zymography as previously described in methods. The results showed a significant elevation in activity of MMP-2 on 80 μM PSH treated NPC cells with exposure to U0126 or SP600125 when compared with PSH treatment only (Figures 4D–G). In addition, a combined treatment of PSH with U0126, but not SP600125, also significantly contributed to the increased expression of MMP-2 level on both cell lines (Figures 4H–K). Therefore, the phosphorylation of ERK1/2 was involved in the regulation of MMP-2 in NPC cells with PSH administration.

### PSH Suppressed EMT in NPC Cells via the MAPK Signaling Pathways

**Figure 5:**
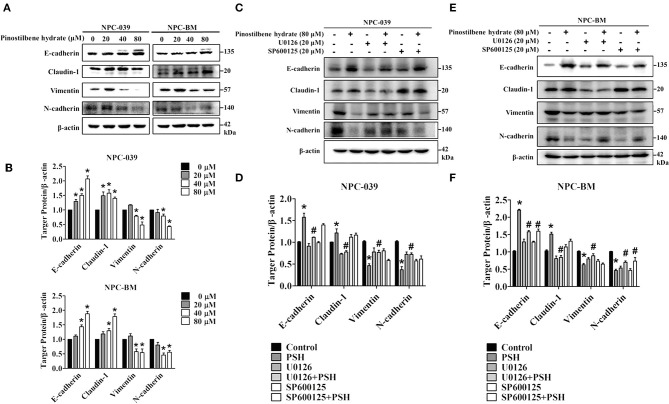
PSH suppressed EMT on NPC cells via the MAPK signaling pathways. **(A,B)** Cells were treated with PSH (0–80 μM) for 24 h. The expression levels of epithelial marker proteins (E-cadherin, Claudin-1) and mesenchymal marker proteins (vimentin, N-cadherin) were analyzed by Western blot. PSH upregulated the expression of E-cadherin and Claudin-1 while downregulating that of N-cadherin and vimentin on both NPC cell lines. ^*^P < 0.05, compared with the control (0 μM). **(C,D)** Combined treatment of PSH with U0126 in NPC-039 cells showed significant increased levels of vimentin and N-cadherin with decreased levels of E-cadherin and Claudin-1 when compared with 80 μM PSH treatment only group. ^*^P < 0.05, compared with the control. ^#^*P* < 0.05, compared with 80 μM PSH treated only group. **(E,F)** Relative to the group treated with 80 μM PSH alone, a co-treatment of PSH with U0126 or SP600125 in NPC-BM cell lines elevated the expression levels of mesenchymal markers and suppressed the expression of epithelial marker proteins. ^*^P < 0.05, compared with the control. ^#^*P* < 0.05, compared with 80 μM PSH treated only.

EMT is also one important process in cancer metastasis. We thus examined the influence of PSH on EMT markers in NPC cell lines. After incubation with various concentrations of PSH (0, 20, 40, and 80 μM) for 24 h, the expression levels of epithelial marker proteins (E-cadherin, Claudin-1) and mesenchymal marker proteins (vimentin, N-cadherin) were analyzed by Western blot assay (Figures 5A,B). The findings indicated that PSH upregulated the expression levels of E-cadherin and Claudin-1 while downregulating that of N-cadherin and vimentin on both NPC cell lines (^*^P < 0.05). To further clarify the association between PSH-suppressed EMT and MAPK signaling pathways, cells were pretreated with or without U0126 or SP600125 for 1 h followed by treatment with PSH (0 and 80 μM) for 24 h. The EMT markers were then determined again. In NPC-039 cells, co-treatment with PSH and U0126 showed significant elevated levels of vimentin and N-cadherin with decreased levels of E-cadherin and Claudin-1 as compared with 80 μM PSH treatment only (Figures 5C,D). Similar results were also noted in NPC-BM cells; co-treatment of PSH with U0126 or SP600125 increased the expression levels of mesenchymal marker proteins while suppressing the levels of epithelial marker proteins relative to the group treated with 80 μM PSH alone (Figures 5E,F). These data revealed that PSH could suppress EMT in human NPC cells through the MAPK pathways.

## Discussion

NPC is a radiosensitive tumor and definitive radiotherapy or chemoradiotherapy forms the main treatment for non-metastatic NPC. The introduction of IMRT and the addition of systemic agents to radiotherapy partly contribute to the improvement in disease control and survival (12, 14). However, the great potentiality of systemic dissemination in NPC patients is troublesome and the outcomes for metastatic cases are heterogeneous and can be very poor (12, 15). In previous study, the anti-metastatic effect of PSH on human oral SCC by downregulation of MMP-2 through MAPK pathways was reported (33). Here, we further examined the effects of PSH on two NPC cell lines and discussed the possible mechanisms. Our results suggested that PSH inhibited the migration and invasion of NPC cells by downregulating MMP-2 expression and suppressing EMT through the MAPK signaling pathways.

Cancer metastasis is a complicated process; both EMT and the invasion of ECM play crucial roles during the metastatic dissemination (17, 18). The MMPs are proteinases that involve the ECM invasion, cancer migration and signaling pathways that control cell growth and angiogenesis (35, 36). Among MMPs, MMP-2 and MMP-9 are collagenase that can selectively degrade type IV collagen, a major component of ECM; overexpression of MMP-2 or MMP-9 is associated with more lymph node metastases, distant metastases and poor survival in head and neck SCC (21–24). Furthermore, Wong and colleague reported that a high pro-MMP2 level was correlated with poor survival in patients with undifferentiated NPC (38). Liu et al. demonstrated an increased MMP-9 level as an unfavorable prognostic factor for NPC; patients with elevated MMP-9 level had a significantly shorter overall survival (OS) time and higher MMP-9 level was positively associated with the status of lymph node metastasis and clinical stage (39). Therefore, MMPs inhibitor had become one of molecular targeted therapy in development for treatment of NPC (40). One study had shown zoledronic acid (MMP inhibitor) combined with chemotherapy could improve progression-free survival (PFS) and OS in NPC patients with bone metastases (41).

In the present study, the cell viability of NPC-039 and NPC-BM cells were significantly decreased after exposure to 80 μM of PSH for 24 h (Figures 1B,C). A significant reduction in migrated distance was also observed through the wound healing assay; PSH markedly suppressed the wound gap closure in a dose-dependent manner (Figures 2A–D). In addition, the ability of cancer cell migration and invasion were inhibited by PSH on both NPC cell lines (Figures 2E–H). These results indicated that PSH played a negative role in the migration and invasion of human NPC cells and PSH could be a potential anti-metastatic drug for NPC. Various studies had shown the anti-metastatic effects of certain agent on NPC or oral cancers through the downregulation of MMP-2 or MMP-9 via the MAPK signaling pathways (33, 34, 37, 42). We thus used the gelatin zymography assay and Western blot to analyze the effects of PSH on MMPs in NPC cells. Our findings showed PSH reduced the activity and the expression of MMP-2 in a dose-dependent manner on NPC cells (Figure 3). We then further evaluated the mechanisms that involved PSH mediated MMP-2 expression in NPC. The levels of MAPK pathway proteins were examined after treatment with PSH. Our data revealed PSH promoted the expression of p-ERK1/2 and p-JNK1/2, but not p-AKT and p-p38, in human NPC cells (Figures 4A–C). Moreover, co-treatment with PSH and ERK1/2 inhibitor (U0126) or JNK1/2 inhibitor (SP600125) contributed to the upregulated activity of MMP-2 in NPC when compared with PSH treatment only group (Figures 4D–G). Therefore, MAPK signaling pathways were involved in PSH mediated MMP-2 regulation in NPC and blocking the ERK or JNK pathway might interfere the anti-metastatic ability of PSH to NPC cells.

EMT is also recognized as an important step in the metastasis of malignancies and the level of E-cadherin was negatively correlated with the clinical prognosis in NPC (40, 43–45). Nowadays demethylation of E-cadherin gene is considered to be a potential therapeutic strategy for patients with NPC (46). Some studies demonstrated the inhibitory effects on migration and invasion of NPC cells by suppressing EMT (47, 48). We then observed the influence of PSH on EMT markers in NPC-039 and NPC-BM cells by Western blot assay. After exposure to PSH, expression levels of the epithelial markers E-cadherin and Claudin-1 increased, while that of the mesenchymal markers N-cadherin and vimentin decreased (Figures 5A,B). Furthermore, combined treatment with PSH and U0126 or SP600125 showed significantly elevated levels of mesenchymal markers with decreased levels of epithelial markers relative to the group treated with PSH alone (Figures 5C–F). Taken together, PSH suppressed the EMT process in NPC cells; the addition of ERK 1/2 inhibitor or JNK1/2 inhibitor reversed this ability and induced NPC cells to regain EMT characteristics which restored the invasiveness of NPC cells. These data suggested that PSH might suppress EMT in human NPC cell lines through the MAPK pathways.

Our results demonstrated that PSH downregulated the activity and the expression of MMP-2 on both NPC-039 and NPC-BM cell lines. A significant reduced migrated distance under the wound healing assay and marked inhibitory effects on the migration and invasion of NPC cells were also observed. After exposure to PSH, the expression of epithelial markers increased, while that of mesenchymal markers decreased, which suggested the ability of PSH to reverse the EMT process in NPC. We also found the associations of MAPK pathways with PSH mediated MMP-2 downregulation and EMT suppression in NPC; ERK and JNK pathways were involved in these molecular mechanisms. In conclusion, PSH inhibited the migration and invasion of human NPC cells *in vitro* by downregulating MMP-2 and suppressing EMT through MAPK signaling pathways. Data obtained from our study indicated that PSH could be a potential anti-metastatic agent for patients with NPC, though more detailed studies may be needed for further application.

## Data Availability Statement

All datasets generated for this study are included in the article/supplementary material.

## Author Contributions

P-YT, M-JH, and M-KC: study conception and design. C-CL, Y-CC, Y-SL, and Y-TH: acquisition, analysis, and interpretation of data. P-YT, Y-TL, and M-JH: drafting/revision of the work for intellectual content and context. M-KC: final approval and overall responsibility for the published work. All authors read and approved the final manuscript.

### Conflict of Interest

The authors declare that the research was conducted in the absence of any commercial or financial relationships that could be construed as a potential conflict of interest.

## Abbreviations

NPC: Nasopharyngeal carcinoma
PSH: Pinostilbene hydrate
ERKs: Extracellular signal–regulated kinases
JNKs: c-Jun N-terminal kinases
MAPK: Mitogen-activated protein kinases.
